# Editorial: The Biomechanics of Competitive Gait: Sprinting, Hurdling, Distance Running and Race Walking

**DOI:** 10.3389/fspor.2021.790934

**Published:** 2021-11-11

**Authors:** Brian Hanley, Johnny Padulo, Jean Slawinski

**Affiliations:** ^1^Carnegie School of Sport, Leeds Beckett University, Leeds, United Kingdom; ^2^Department of Biomedical Sciences for Health, Faculty of Medicine and Surgery, University of Milan, Milan, Italy; ^3^Institut National du Sport, de l'Expertise et de la Performance (INSEP), Paris, France

**Keywords:** track athletics, endurance exercise, sport performance, sport technology, sprint speed

This Research Topic features each aspect of competitive gait, comprising sprinting, hurdling, distance running, and race walking. Each of these forms of gait has its own biomechanical signature, and the research published was carried out by 31 authors across 11 countries to provide modern, relevant and robust practical applications to athletes, coaches, scientists, and others who are involved in competitive gait at all standards.

One of the best known running events held across the globe is the marathon, with many athletes of all abilities taking part and millions more watching live broadcasts. Performances in the marathon have improved in recent years, with Bermon et al. examining the role of advanced shoe technology on elite athletes' finishing times over the 42.2 km distance, as well as in other distance races. They found a large improvement in running times over the past few years and attributed much of this to the contribution of new shoe design. Another study on the marathon analyzed the differences in performance between athletes who land with a rearfoot strike, and those who land on the midfoot or forefoot (Hanley, Bissas et al.). They found that although there were differences between the biomechanics of athletes with each footstrike pattern, these were quite small and didn't seem to affect overall performance. Hanley, Bissas et al. also examined the effects of fatigue on marathon gait and differences between men and women for the most in-depth study of elite marathon kinematics ever conducted.

Changes in gait with fatigue, this time within a group of middle-distance runners, was also the focus of the study by Möhler et al.. These authors showed that these runners changed their stance time, rather than step frequency or step length, to maintain a constant running speed during treadmill testing. The study went further than previous research by using the analysis of both discrete parameters and time series analysis in 3D, and provided valuable practical applications for coaches. In another treadmill study using 3D data collection methods, Sundström et al. examined how runners adapt their lower-limb movement patterns on downhill slopes. The authors found that runners changed their hip movement as speed increased, but made modifications to knee kinematics when responding to changes in slope. It was observed that running economy was better at moderate speeds than near-race speed on steep downhill slopes (−10°), whereas the reverse was true on less-steep declines (−5°), and so running downhill in races should be completed at a slower pace to retain metabolic energy. Of course, biomechanists who work in competitive gait are not just concerned with improving performance, but also with reducing the risk of injury. In their study on mild leg length discrepancy, Menez et al. found that orthotic insoles can improve gait symmetry, particularly in the pelvis and ankle, and reduce immediate pain, which is an important finding for those who could benefit from a relatively straightforward intervention.

Analyzing performance in competition, when athletes are in their most natural setting, is rarely conducted because of the difficulty of obtaining *in-vivo* measurements. For this reason, several methods for calculating or estimating important variables have been developed. For analyzing the 100 m sprint, Seidl et al. reported on a pilot study conducted under field conditions, finding that a method using radio-based position detection obtained valid results for spatiotemporal variables such as step length and step time, and suggested that the development of this approach could allow for valuable measurements in competition. In their study using shoe-worn inertial sensors, Falbriard et al. estimated overground running speed and compared these sensors with Global Navigation Satellite System (GNSS) technology. They took three approaches to extracting the data and found that the limitations of direct estimation of foot velocity and a general linear model could be overcome with a personalized online model. Mercier et al. took a modeling approach to analyzing strategy and optimization of performance in the 10,000 m event by applying a simulation to real athletes' time split data collected at the 2017 IAAF World Championships. By doing so, they were able to show the negative effect of the bends on speed maintenance and how a conservation of anaerobic energy in the middle of the race allows for a faster last lap.

Two of the most technical events in competitive gait are hurdling and race walking. These forms of gait are restricted, in hurdling by the position and height of the 10 barriers, and in race walking by the need to straighten the knee and avoid visible loss of ground contact. This makes the analysis of technique using biomechanical methods highly relevant for understanding how improvements in performance can be achieved. In their study of the world's best hurdlers competing in the 2017 IAAF World Championship finals, Hanley, Walker et al. used high-speed cameras to compare men's and women's techniques. They found that the lower relative hurdle heights in the women's event result in a less demanding task, and mean that the techniques adopted by men and women are not the same. The men's hurdle is so much higher for them relative to their height that it affects their vertical movement to a greater extent and is more disruptive of forward momentum. Gravestock et al. also analyzed men and women in their study of the role of upper body biomechanics in elite race walkers, but they found little difference in how men and women achieve their gait mechanics. Within the group, pelvic girdle movements were very important in optimizing spatiotemporal variables, and other torso movements were made in reaction to the absence of knee flexion during midstance. Gravestock et al. found through their use of electromyography that there was no evidence that muscle strength was important for better race walking, and so they instead recommended the development of resistance to fatigue in this endurance event.

In conclusion, the articles that have contributed to this Research Topic have covered the main areas in competitive gait. We hope that these novel studies will aid those working in this area in developing performance across a range of athletic abilities. These studies will form a basis for future research that should continue to develop our scientific knowledge in this most popular sport.

## Author Contributions

All authors listed have made a substantial, direct and intellectual contribution to the work, and approved it for publication.

## Conflict of Interest

The authors declare that the research was conducted in the absence of any commercial or financial relationships that could be construed as a potential conflict of interest.

## Publisher's Note

All claims expressed in this article are solely those of the authors and do not necessarily represent those of their affiliated organizations, or those of the publisher, the editors and the reviewers. Any product that may be evaluated in this article, or claim that may be made by its manufacturer, is not guaranteed or endorsed by the publisher.

